# A comprehensive mouse brain acetylome-the cellular-specific distribution of acetylated brain proteins

**DOI:** 10.3389/fncel.2022.980815

**Published:** 2022-08-30

**Authors:** Yuhua Ji, Zixin Chen, Ziqi Cen, Yuting Ye, Shuyuan Li, Xiaoshuang Lu, Qian Shao, Donghao Wang, Juling Ji, Qiuhong Ji

**Affiliations:** ^1^Department of Immunobiology, College of Life Science and Technology, Jinan University, Guangzhou, China; ^2^Department of Neurology, Affiliated Hospital of Nantong University, Nantong, China; ^3^Department of Pathology, College of Medicine, Nantong University, Nantong, China

**Keywords:** brain, acetylome, cellular-specific distribution, neuron, cerebrovascular endothelial cell, mouse

## Abstract

Nε-lysine acetylation is a reversible posttranslational modification (PTM) involved in multiple physiological functions. Genetic and animal studies have documented the critical roles of protein acetylation in brain development, functions, and various neurological disorders. However, the underlying cellular and molecular mechanism are still partially understood. Here, we profiled and characterized the mouse brain acetylome and investigated the cellular distribution of acetylated brain proteins. We identified 1,818 acetylated proteins, including 5,196 acetylation modification sites, using a modified workflow comprising filter-aided sample preparation (FSAP), acetylated peptides enrichment, and MS analysis without pre- or post-fraction. Bioinformatics analysis indicated these acetylated mouse brain proteins were mainly located in the myelin sheath, mitochondrial inner membrane, and synapse, as well as their involvement in multiple neurological disorders. Manual annotation revealed that a set of brain-specific proteins were acetylation-modified. The acetylation of three brain-specific proteins was verified, including neurofilament light polypeptide (NEFL), 2’,3’-cyclic-nucleotide 3’-phosphodiesterase (CNP), and neuromodulin (GAP43). Further immunofluorescence staining illustrated that acetylated proteins were mainly distributed in the nuclei of cortex neurons and axons of hippocampal neurons, sparsely distributed in the nuclei of microglia and astrocytes, and the lack of distribution in both cytoplasm and nuclei of cerebrovascular endothelial cells. Together, this study provided a comprehensive mouse brain acetylome and illustrated the cellular-specific distribution of acetylated proteins in the mouse brain. These data will contribute to understanding and deciphering the molecular and cellular mechanisms of protein acetylation in brain development and neurological disorders. Besides, we proposed some problems that need to be solved in future brain acetylome research.

## Introduction

Nε-lysine acetylation is a reversible posttranslational modification (PTM). The dynamic balance of protein acetylation is mainly regulated by two types of enzymes: lysine acetyltransferases (KATs or HATs) that transfer acetyl groups from CoA to the protein N ε-lysine, and lysine deacetylases (KDACs or HDACs) that act as “erasers” (Marmorstein and Zhou, 2014). Histones are the first identified acetylated proteins (Allfrey et al., 1964). With the advancement of MS-based proteomics, thousands of acetylated proteins have been identified (Yang et al., 2011; Kosanam et al., 2014; Holper et al., 2015; Lee et al., 2016; Xie et al., 2018). Meanwhile, the knowledge of the biological functions of protein acetylation has expanded from their initial roles in epigenetics through the regulation of histone acetylation to more extensive biological processes by modulating protein-protein interactions, enzyme activity, and protein subcellular localization and stability (Narita et al., 2019).

The critical roles of protein acetylation in brain development and function have been well-acknowledged. Genetic studies advised the close relationship between the mutations of HATs and HDACs and human neurodevelopmental and psychiatric disorders (Petrij et al., 1995; Williams et al., 2010; Clayton-Smith et al., 2011; Harakalova et al., 2012). Moreover, HATs and HDACs mutation mouse models studies confirmed the involvement of protein acetylation in brain development and disorders and highlighted the essential roles of protein acetylation in modulating proliferation and differentiation of neural stem cells, maturation of astrocytes, oligodendrocytes, and microglia, and the establishment of neuronal circuits (Volmar and Wahlestedt, 2015; Tapias and Wang, 2017). More importantly, targeting HDACs by pan and selective HDAC inhibitors (HDACis) has emerged as a highly promising therapeutic strategy for multiple neurological disorders, such as neurodegenerative diseases (Chuang et al., 2009; Ziemka-Nalecz et al., 2018; Shukla and Tekwani, 2020), psychiatric disorders (Abel and Zukin, 2008), and acute brain injury (Yang et al., 2021). Impressively, HDACi treatment can target almost all known pathways involved in the complex course of these neurological disorders, ranging from attenuation of cell death, suppression of inflammatory processes, and enhanced blood flow to the stimulation of repair mechanisms and increased plasticity (Langley et al., 2009; Didonna and Opal, 2015; Shukla and Tekwani, 2020). However, the cellular and molecular mechanisms underlying the roles of HATs and HDACs in brain development and functions and the multifacet effects of HDACi treatment on various neurological disorders are still partially understood.

A challenge for such studies is the complexity of protein acetylation networks regulated by HATs and HDACs (Nalawansha et al., 2018; Weinert et al., 2018). Thus, screening the pathophysiological relevant acetylated proteins by investigating the effects of HAT and HDAC mutation and HDACis on brain acetylome will significantly contribute to our deciphering and understanding of the mechanism underlying the roles of protein acetylation in brain development and various neurological disorders. However, our knowledge of brain acetylome and its features is still minimal. So far, the most comprehensive brain acetylome was reported by Lundby, A et al., who identified 1,653 acetylated proteins with 4,782 modification sites in the rat brain (Lundby et al., 2012).

Mice have been the most common choice for modeling neurodegenerative diseases (Fisher and Bannerman, 2019; Coleman and Höke, 2020). Here, we established a comprehensive mouse brain acetylome using a modified workflow in which the acetylated peptides were enriched and analyzed by MS without pre- or post-fraction. Besides comprehensively characterizing the obtained mouse brain acetylome, we analyzed the cellular and subcellular distribution of the acetylated proteins in the mouse brain. In addition, given the significant differences observed between the present mouse brain acetylome and the previous rat brain acetylome (Lundby et al., 2012), we discussed the potential reasons for this discrepancy and proposed some technical challenges in future acetylome research.

## Materials and methods

### Animals

The experimental protocols and procedures involving animals and their care were conducted according to the National Institutes of Health Guide for Care and Use of Laboratory Animals. The Administration Committee of Experimental Animals of Jinan University approved the experimental protocol. Male C57BL/6 wild-type mice (10 weeks old) provided by the Guangdong medical laboratory animal center were raised at 25^°^C, 40–60% relative humidity. Animals were allowed access to food and water *ad libitum*.

### Mouse tissue protein extraction

Mice were euthanasia with 5% isoflurane (RWD, Shenzhen) and perfused intracardially with 30 ml cold PBS. Then, the brain, liver, heart, and spleen were homogenized in RIPA lysis buffer (Beyotime Biotechnology, P0013B) or NP-40 lysis buffer (Beyotime Biotechnology, P0013F). For proteome and acetylome analysis, mice brains were homogenized in urea lysis buffer (8 M sequencing grade urea, 100 mM Tris-HCl PH 8.0). Protease inhibitors cocktail tablets (Roche Diagnostics GmbH, Mannheim, Germany) were added to all three protein lysis buffers. Centrifuge the lysis at 10,000 g for 15 min at 4°C, and the supernatant was collected and stored at -80°C. Protein concentration was determined using a BCA Protein Assay Kit (Thermo Fisher Scientific, Pierce BCA Protein Assay Kit, Cat#23225).

### Immunoprecipitation

Proteins extracted by NP-40 lysis buffer (Beyotime Biotechnology) from the mouse brain were incubated with anti-acetyllysine antibody conjugated agarose beads (PTM-104, PTM Biolab) overnight at 4°C. After washing the beads thrice with NP-40 lysis buffer, the proteins were eluted by boiling in 20 μl of 2 × SDS loading buffer for 5?min. After brief centrifugation at 10,000 g, the supernatant was collected and stored at -80°C.

### Western blotting

Proteins extracted by RIPA lysis buffer and the samples from immunoprecipitation were separated by 12% SDS-PAGE and electro-blotted onto the PVDF membrane (Millipore, United States). Membranes were then blocked with 1% BSA for one hour and then incubated with the following primary antibodies overnight at 4°C: anti-acetyllysine antibody (1:5000 (V/V), PTM-101, PTM Biolab, China), CNPase rabbit monoclonal antibody (1:1000 (V/V), 5664, Cell Signal Technology), NEFL rabbit monoclonal antibody (1:500, 12998-I-AP, Proteintech, United States), and Gap 43 rabbit polyclonal antibody [1:1,000 (V/V), 16971-I-AP, Proteintech, United States]. Next, the membranes were washed three times with PBST. The membranes were then incubated with horseradish peroxidase-conjugated secondary antibody [1:1,000 (V/V), Horse anti-mouse (7076S), Goat anti-rabbit (7074S), Cell Signal Technology], and for one hour at room temperature (RT). After washing, ECL (P0018FM, Beyotime Biotechnology, China) was added to visualize the blotted bands.

### Protein digestion and acetylated peptides enrichment

Equal amounts of protein from the brains of three mice were pooled. It has been proposed that pooling the samples from different biological sources can reduce biological variation (Diz et al., 2009). Proteins were digested by Filter-aided sample preparation (FASP) (Wisniewski et al., 2009). In brief, four milligrams of protein were transferred to an ultrafiltration tube (Ultracel-10K, Millipore, United States). The protein samples were reduced with a 20 mM dithiothreitol (DTT) for 1.5 h at 37°C and then alkylated with 100 mM iodoacetamide (IAA) for 20 min at RT in the dark. Then, the processed samples were digested with Trypsin [1:100 (W/W), V5111, Promega, United States], The resulting peptide mixtures were purified by C18 SPE columns (Agilent, United States) and freeze-dried.

The enrichment of acetylated peptides was performed according to the manufacturer’s instructions. In brief, the freeze-dried peptides were dissolved in NETN buffer (100?mM NaCl, 1?mM EDTA, 50?mM Tris-HCl, 0.5% NP-40, and pH 8.0) and incubated with pre-washed anti-acetyllysine antibody beads (PTM-104, Biolab, China) overnight with gentle shaking at 4?°C. After washing five times with NETN buffer and twice with ddH_2_O, the bound peptides were eluted with 1% trifluoroacetic acid.

### Liquid chromatography tandem mass spectrometry analyses and MS data processing

The purified peptide or enriched acetylated peptide samples were separated by an EasyNano LC1000 system (San Jose, Thermo Fisher Scientific) using a C18 column (3 μm, 75 μm × 15 cm) at a 500 nl/min flow rate. A 75-min gradient was set as follows: 1% B (0.1% FA in ACN)/99% A (0.1% FA in H_2_O) to 3% B in 2 min, 3% B to 8% B in 8 min, 8% B to 20% B in 45 min, 20% B to 30% B in 12 min, 30% B to 90% B in 1 min and kept for 7 min. MS data were acquired with a data-dependent acquisition mode using Orbitrap Fusion Lumos (Bremen, Thermo Fisher Scientific). A full-speed scan mode of 3 seconds with an MS1 scan range of m/z 350-1550 was used for the data acquisition. The other parameters were set as below: MS1 and MS2 resolution were set to 120 and 30 K; Automatic gain control (AGC) was used to prevent overfilling of the ion trap, and the value for MS1 and MS2 were 1e6 and 5e4, respectively; isolation window was 1.6 m/z, higher energy C-trap dissociation (HCD) with normalized collision energy (NCE) was 32, dynamic exclusion time was 20 s.

Raw MS data were searched against a *Mus musculus* protein database (Uniprot, 86,430 entries, acquired on 2017.4.26) using Proteome Discoverer 2.1 (Thermo Fisher Scientific, San Jose, CA, United States). The following parameters were used for data processing: trypsin, with a maximum number of three missed cleavages; precursor and fragment ion mass tolerance was set to 10 ppm and 0.02 Da; variable modification was set to Oxidation on methionine (M, 15.9949), deamidation on glutamine and asparagine (N/Q, 0.9840/0.9847) and acetylation on lysine (K, 42.0106) and protein N-terminus; fixed modification was set to Carbamidomethylation on cysteine (C, 57.0215); An algorithm of Percolator (Spivak et al., 2009) was used to keep peptide FDR less than 1% and the q-value used for protein identification was less than 0.01. Modification probability was kept at more than 0.75.

### Bioinformatics analysis

IceLogo software (version: 1.3.8) (Colaert et al., 2009) was adopted to analyze the Kac Motif. The IceLogo built-in *Mus musculus and Rattus norvegicus* databases were used as background. The results were presented as fold change, and *p* < 0.01 was considered statistically significant. The Gene Ontology Consortium bioinformatics functional annotation tool R package Clusterprofile (v3.14.3) and mouse and rat database (org.Mm.eg.db or org.Rn.eg.db, version: 2.1) were used to identify enriched biological processes (BP) and cellular component (CC) terms of acetylated proteins (Yu et al., 2012). Kyoto Encyclopedia of Genes and Genomes (KEGG) analysis (Yu et al., 2012) was carried out to infer the biological pathways involved in these acetylated proteins. The GO enrichment and KEGG pathway analysis results were illustrated as dot plots by R package ggplot2 (Villanueva and Chen, 2019).

### Immunofluorescent staining

For immunostaining, euthanized mice were perfused intracardially with 30 ml pre-cold PBS and 20 ml 4% paraformaldehyde (PFA) sequentially. After post-fixation, the brains were cut coronally at 50 μm thickness by a vibratome (Leica, Buffalo Grove, IL, United States). Sections were permeabilized in PBST (0.1% Triton X-100 in PBS) for 10 min, blocked for one hour at RT in blocking buffer (10% BSA in PBS), and incubated overnight at 4^°^C with the following primary antibodies: a mouse monoclonal anti-acetyllysine antibody (1:500, PTM101, PTM Biolab, Hangzhou, China), a rabbit monoclonal anti-NeuN antibody (1:250, D3S3I, Cell Signaling Technology), a rabbit polyclonal anti-Iba-1 antibody (1:1000, 019–19,741, Wako Chemicals GmbH, Neuss, Germany), a rabbit monoclonal anti-GFAP antibody (1:250, Cell Signaling Technology), and a rabbit polyclonal anti-CD31 antibody (1:100, 557355, BD Biosciences, United States). The sections were then incubated with the following secondary antibodies for 2 h at RT: Alexa Fluor 488-conjugated goat anti-mouse IgG and Alexa Fluor 555-conjugated 82071553 (1:200, Cell Signaling Technology). Nuclei were counterstained with DAPI (1 μg/ml, Sigma). Images were captured on a Zeiss LSM710 confocal microscope (Munich, Germany).

## Results

### Profiling the mouse brain acetylome using a simplified workflow

**FIGURE 1:**
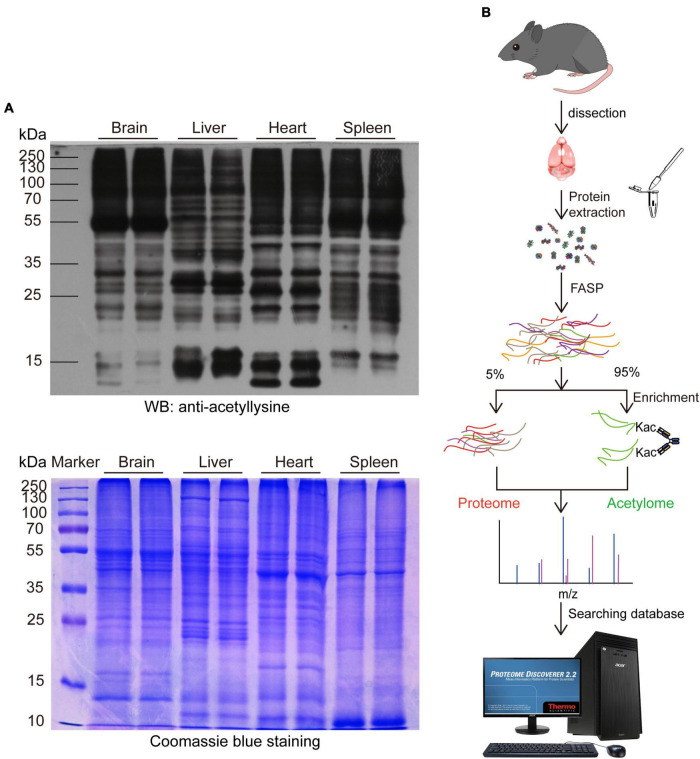
Profiling the mouse brain acetylome by using a simplified workflow. **(A)** Comparing the expression pattern of lysine-acetylated proteins in the mouse brain, liver, heart, and spleen. 30 μg proteins from the mouse brain, liver, heart, and spleen were separated by SDS-PAGE and probed with an anti-acetyllysine antibody (upper). Coomassie brilliant blue-stained SDS-PAGE was used as a loading control (Lower). **(B)** Schematic diagram depicting the simplified workflow for profiling mouse brain acetylome.

We first performed a WB analysis using an anti-acetyllysine antibody to take an overview of the expression pattern of acetylated proteins in the mouse brain and compared it with the liver, heart, and spleen. As shown in Figure 1A, the expression pattern of acetylated proteins in the mouse brain was significantly different from the liver, heart, and spleen. Notably, The molecular weight of most acetylated proteins in the mouse brain was higher than 55 kDa, and those lower than 55 kDa were less acetylated. Moreover, the expression pattern between the proteins and acetylated proteins from the same tissue was distinct.

Then, we profiled the mouse brain acetylome by using a simplified workflow comprising FASP, acetylated peptide enrichment, and MS analysis without pre-fraction. Four milligrams of total mouse brain proteins were digested by FASP protocol, and around 600–800 μg peptides could be obtained after purification. The 95% purified peptides were subjected to the enrichment of acetylated peptides using anti-acetyllysine antibody conjugated beads. Afterward, the enriched peptides and the remaining 5% purified peptides were subjected to a 75 min liquid chromatography tandem mass spectrometry (LC-MS/MS) analysis without fraction (Figure 1B). In the three independent biological replicas, 11,599, 15,009, and 14,357 peptides (FDR < 1%) were identified, comprising 5,476, 7,103, and 5,688 acetylated peptides, respectively (Table 1 and Supplementary Table 1). The enrichment efficiency of this study was greater than 44%, which was slightly higher than the 40% of a previous rat brain acetylome study (Lundby et al., 2012). The percentage of acetylated peptides identified in the unenriched samples was less than 0.19% (Table 1 and Supplementary Table 2). Thus, in this study, the enrichment procedure increased the percentage of acetylated peptides in the samples by over 200 times.

**TABLE 1:** Statistics of acetylome and proteome of the C57BL/6 mouse brain.

	Acetylome				Proteome			
	Exp1	Exp2	Exp3	Merged	Exp1	Exp2	Exp3	Merged
Identified peptides	11,599	15,009	14,357	40,388	18,547	13,924	20,455	52,977
Acetylated peptides	5,476	7,103	5,688	17,939	30	19	51	100
Enrichment efficiency (%)	47.21	47.32	39.62	44.42	0.16	0.14	0.25	0.19
Unique peptides	7,017	6,910	8,598	12,974	11,002	9,569	10,424	13,513
Unique acetylated peptides	3,095	2,727	3,041	4,972	28	19	46	79
Acetylated sites	3,324	2,886	3,314	5,196	12	7	16	35
Acetylated proteins	1,296	1,372	1,306	1,818	12	7	14	32

**FIGURE 2:**
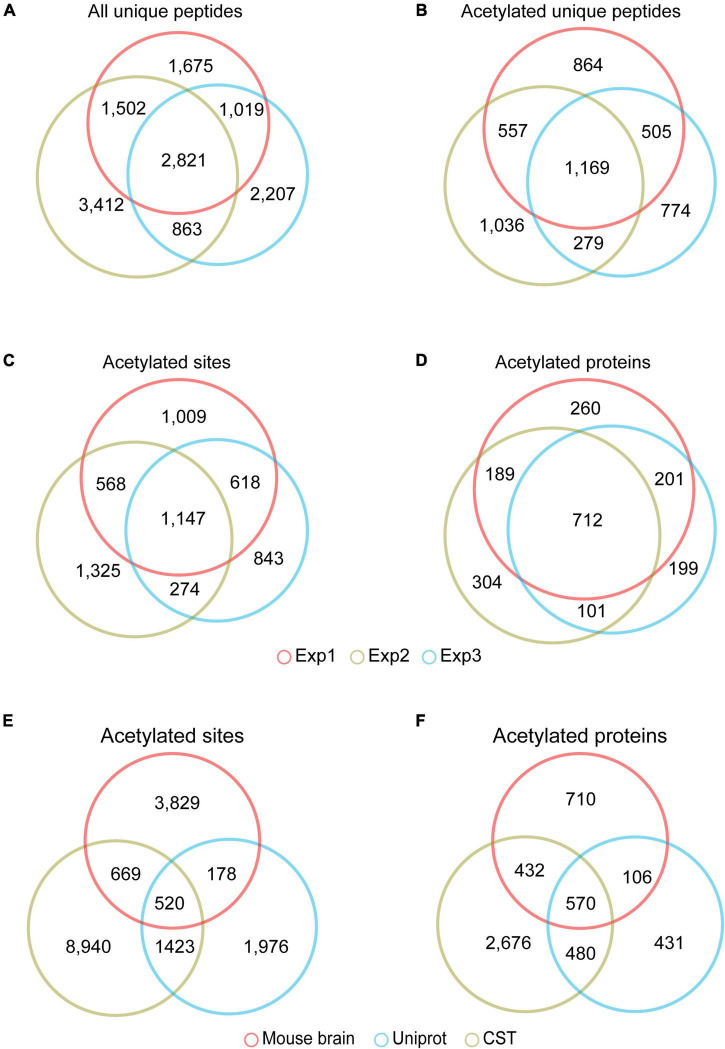
The acetylome of the mouse brain. Venn diagram showed the overlapped unique peptides **(A)**, unique acetylated peptides **(B)**, acetylated proteins **(C)**, and acetylated sites **(D)** among the three independent experiments. Venn diagram showed the overlap of the acetylated lysine sites **(E)** and proteins **(F)** identified in the mouse brain and those in the PTM databases of Uniprot Swiss-Prot database (https://www.uniprot.org) and CST PhosphoSite*^Plus^* (https://www.phosphosite.org).

Among the 7,017, 6,910, and 8,598 unique peptides (Figure 2A), there were 3,095, 2,727, and 3,041 unique acetylated peptides, containing 3,324, 2,886, and 3,314 acetylation sites and corresponding to 1,296, 1,372, and 1,306 acetylated proteins, respectively (Supplementary Table 3). Of these acetylated peptides identified in three replicas, 1,169 overlapped (Figure 2B), corresponding to 1,147 sites (Figure 2C) and 712 proteins (Figure 2D), respectively. To obtain a more comprehensive mouse brain acetylome, we merged the raw MS data from three replicas. After searching against the same database using the same criteria, 1,818 acetylated proteins containing 5,196 acetylated sites were identified (Table 1 and Supplementary Table 3). By Blasting the PTM databases from Uniprot and CST PhosphoSitePlus, we found that 3,829 acetylation sites detected in this study were newly discovered acetylated sites (Figure 2E), corresponding to 710 acetylation proteins (Figure 2F and Supplementary Table 4).

**FIGURE 3:**
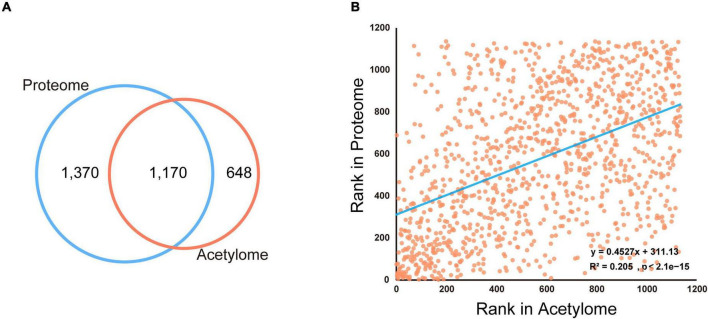
Comparison of the proteome and acetylome of mouse brain. **(A)** Venn diagram showed the overlapped proteins identified in the mouse brain proteome and acetylome. **(B)** The dot plot showed a weak correlation between protein abundance and acetylation modification in the mouse brain.

Venn diagram (Figure 3A) showed that most of these acetylated proteins (1,170 out of 1,818) were identified in the proteome analyzed by the same MS analysis conditions, indicating the majority of the acetylated proteins detected were high abundance proteins. To explore the relationship between protein abundance and protein acetylation in the mouse brain, we ranked these 1,170 proteins shared by brain proteome and acetylome according to their ion intensity, respectively (Supplementary Table 5). The correlation factor between their ranks was 0.205 (Figure 3B). There are two mechanisms for protein acetylation: enzyme-dependent and non-enzyme-dependent (Narita et al., 2019). The weak correlation between protein acetylation modifications and their abundance suggested that the acetylated proteins in the brain were mainly enzyme-dependent modifications.

### Characteristics of mouse brain acetylome

**FIGURE 4:**
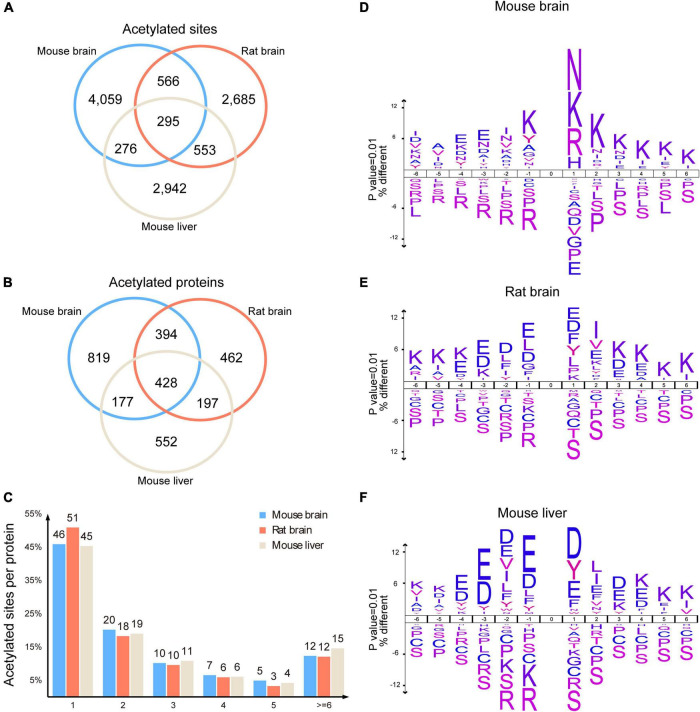
Characteristics of the mouse brain acetylome. Venn diagram showed the shared acetylated sites **(A)** and proteins **(B)** among the acetylomes of the mouse brain, rat brain, and mouse liver. Histogram illustrating the number of acetylated sites per acetylated protein in the mouse brain, rat brain, and mouse liver **(C)**. Representations of the sequence preference flanking the acetylated lysine in the mouse brain **(D)**, rat brain **(E)**, and mouse liver **(F)** (*p* < 0.01). The size of the letter indicated the frequency of the corresponding amino acid residue in that position.

To characterize the mouse brain acetylated proteins, we analyzed their acetylated sites and motif preference and compared them with previous rat brain acetylome (comprising 1,653 acetylated proteins with 4,782 modification sites) (Lundby et al., 2012) and mouse liver acetylome (comprising 1,481 acetylated protein and 4,067 acetylation sites) (Weinert et al., 2013). As expected, we observed significant differences between the protein species of the mouse brain and mouse liver acetylomes (Weinert et al., 2013). Their shared acetylated sites and proteins were only 571 and 605, respectively (Figures 4A,B), reflecting the tissue specificity of acetylated proteins. However, we unexpectedly found that even though the number of acetylated sites and proteins identified in the mouse brain was comparable to that of the rat brain (Lundby et al., 2012), only 861 acetylated sites and 822 acetylated proteins were shared, accounting for 49.7% and 18% of the rat brain acetylome (Figures 4A,B), indicating the significant differences between the present mouse brain acetylome and the previous rat brain acetylome (Lundby et al., 2012).

Statistical analysis of the acetylated sites per protein showed that similar to rat brain acetylome (Lundby et al., 2012), 76% of acetylated proteins in the mouse brain had 1–3 acetylated sites, and 12% of proteins had more than 6 modification sites (Figure 4C). By contrast, more than 15% of proteins in the mouse liver had more than 6 acetylated sites (Weinert et al., 2013). Among these acetylated proteins in the mouse brain, spectrin alpha chain, non-erythrocytic 1 (Sptan1), had the highest number of acetylated sites (59 acetylated sites). In comparison, Carbamoyl-phosphate synthase (1) had the highest acetylated sites (52 acetylated sites) in the mouse liver (Supplementary Table 3).

Using Icelog software (version 1.3.8), we analyzed the preferences of motifs flanking acetylated lysine of brain proteins. In the mouse brain, the acetylation was more likely to occur in proteins with lysine enriched at +1 to +6, and toward positively charged residues at +1, such as lysine (K), arginine (R), and histidine (H), as well as polar amino acid asparagine (N) (Figure 4D). However, in the rat brain, the amino acids surrounding acetylated lysine were mainly negatively charged glutamic acid (E) and aspartic acid (D), along with a small proportion of hydrophobic amino acids phenylalanine (F) and tyrosine (Y) (Figure 4E). Unexpectedly, the preference for motifs of acetylated rat brain proteins was more similar to those in mouse livers (Figure 4F), as both of them comprised extensive distribution of Aspartate (D) and Glutamate (E).

## Bioinformatic analysis of mouse brain acetylome

**FIGURE 5:**
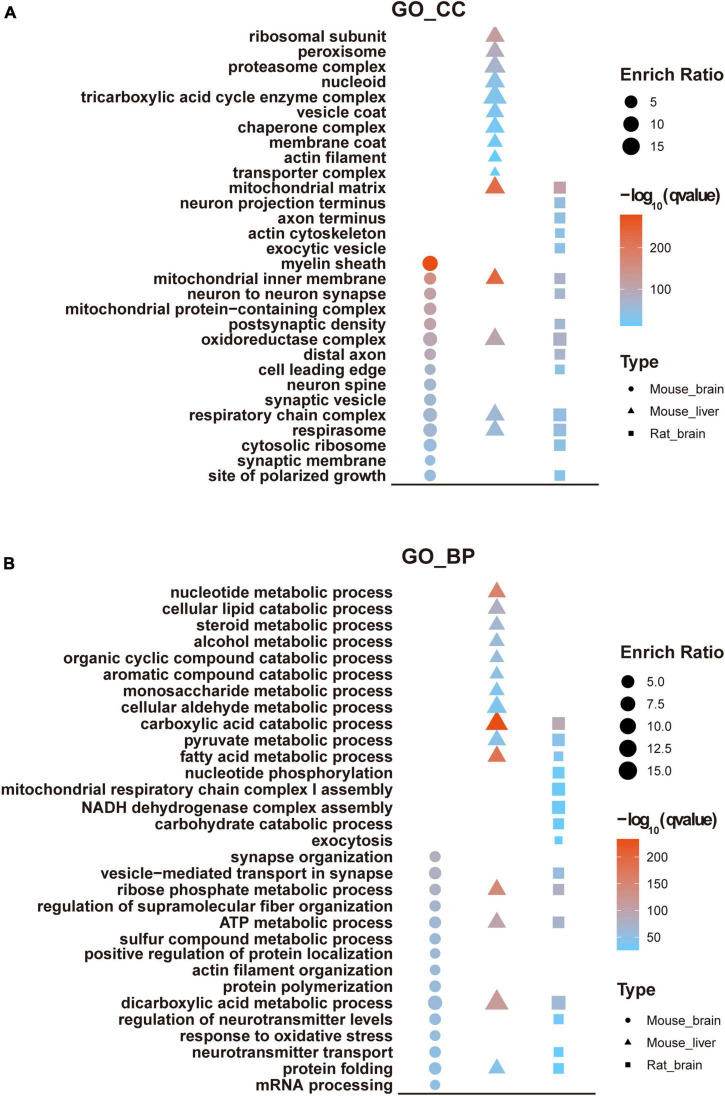
GO enrichment analysis of mouse brain acetylome. GO enrichment analyzed cellular components **(A)** and biological processes **(B)** of the acetylome of the mouse brain and compared them with the rat brain and mouse liver.

We performed GO analysis to examine the enriched functional categories of acetylated proteins in the mouse brain. Meanwhile, the results were compared with mouse liver (Weinert et al., 2013) and rat brain acetylomes (Lundby et al., 2012). Notably, the Cellular component (CC) of acetylated proteins in the mouse brain was mainly located in the myelin sheath, mitochondrial inner membrane, neuron to neuron synapse. In contrast, the acetylated proteins in the mouse liver were mainly located in the mitochondrial inner membrane, mitochondrial matrix, oxidoreductase complex, ribosomal subunit, and peroxisome (Figure 5A). Accordingly, the biological processes (BP) involved in the synapse organization were significantly enriched in the mouse brain acetylome. In contrast, those acetylated proteins in the mouse liver were significantly enriched in metabolic biological processes, including the carboxylic acid catabolic and fatty acid metabolic processes (Figure 5B). These discrepancies mainly reflected the tissue specificity. However, we found apparent differences between mouse and rat brain acetylome (Lundby et al., 2012) in the GO analysis results. For instance, in rat brain acetylome, the mitochondrial matrix was the most enriched category of cellular components rather than the myelin sheath in mouse brain acetylome.

**FIGURE 6:**
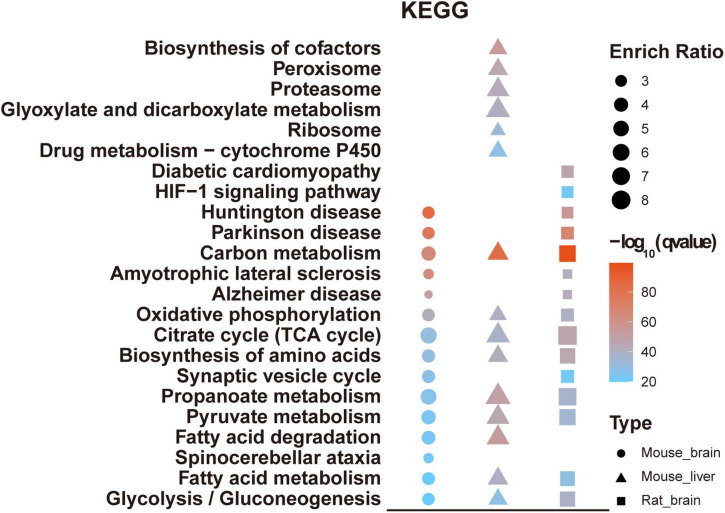
KEGG analysis of mouse brain acetylome. The KEGG pathway analyzed the mouse brain acetylome and compared it with the rat brain and mouse liver.

KEGG analysis suggested that pathways such as carbon metabolism, oxidative phosphorylation, citrate cycle, and pyruvate metabolism were significantly enriched in all three tissues, consistent with the critical roles of protein acetylation in modulating energy metabolism (Rodgers et al., 2008; Zhao et al., 2010). In addition, multiple pathways associated with neurological diseases were significantly enriched in the mouse and rat brain acetylomes, such as synaptic vesicle cycle, Huntington’s disease, Parkinson’s disease, and Alzheimer’s disease, while the pathways significantly enriched in mouse liver acetylome were the biosynthesis of the cofactor, peroxisome, and proteasome (Figure 6).

## Manual annotation and verification of acetylated mouse brain proteins

**FIGURE 7:**
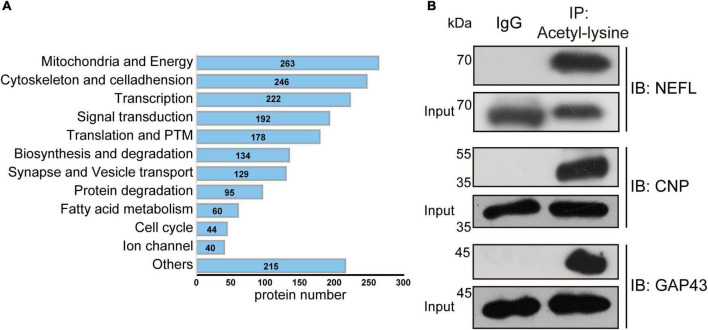
Manual annotation and verification of acetylated modified proteins in the mouse brain. **(A)** The bar chart depicted the 12 categories of acetylated proteins classified by manual annotation. The number indicated the acetylated proteins in each category. **(B)** Verification of newly identified acetylated modified mouse brain proteins. Total acetylated proteins from brain extracts were immunoprecipitated by anti-acetyllysine antibody conjugated beads and probed with antibodies against the indicated proteins.

To gain more insight into the biological functions of acetylated proteins in the mouse brain, we classified these identified acetylated proteins into 12 categories according to their main biological functions by Uniprot database and literature search (Figure 7A and Supplementary Table 6). The most important finding was that many brain- or neuron-specific proteins were modified by acetylation, such as cytoskeletal components, synaptic components, and ion channel proteins (Table 2). There are 105 acetylated mouse brain-specific proteins in Table 2, comprising 369 acetylation sites, among which 60 proteins and 49 acetylation sites have been reported in rat brains (Lundby et al., 2012), accounting for 57.14 and 13.28%, respectively.

**TABLE 2:** The acetylated modified brain-specific or enriched proteins.

Accession	Protein description	Gene name	AcK sites
**Cytoskeleton and cell adhesion**			
Q8BIZ1	Ankyrin repeat and sterile alpha motif domain-containing protein 1B	Anks1b	Acetyl [K1086]
Q9ES28	Rho guanine nucleotide exchange factor 7	Arhgef7	Acetyl [K421]
P28658	Ataxin-10	Atxn10	Acetyl [K384]
Q9Z0H8	CAP-Gly domain-containing linker protein 2	Clip2	Acetyl [K860]
Q8BH44	Coronin-2B	Coro2b	Acetyl [K38; K471]
Q3UPX0	Ketimine reductase mu-crystallin	Crym	Acetyl [K54]
Q02248	Catenin beta-1	Ctnnb1	Acetyl [K180; K181]
O35927	Catenin delta-2	Ctnnd2	Acetyl [K371]
Q8CHG1	MKIAA0369 protein (Fragment)	Dclk1	Acetyl [K106; K107; K284]
Q8BKX1	Brain-specific angiogenesis inhibitor 1-associated protein 2	Baiap2	Acetyl [K36; K142; K143; K206; K351]
E9QM99	Dedicator of cytokinesis protein 10	Dock10	Acetyl [K672]
A0A0R4J1N0	Dihydropyrimidinase-related protein 4	Dpysl4	Acetyl [K41; K289]
Q5EBJ4	Ermin	Ermn	Acetyl [K167]
E0CXB9	Alpha N-catenin	Ctnna2	Acetyl [K132; K693; K695; K707; K749; K759; K760; K920]
E0CZ72	Kinesin-like protein	Kif2a	Acetyl [K101; K259; K261]
Q80TH1	MKIAA1232 protein (Fragment)	Dlg3	Acetyl [K586; K850; K862]
Q8C854	Myelin expression factor 2	Myef2	Acetyl [K512]
O08553	Dihydropyrimidinase-related protein 2	Dpysl2	Acetyl [ 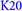 ; K254; K258; K293; K297; K390; K423; K451; 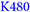 ; K511; K520]
E9PWE8	Dihydropyrimidinase-related protein 3	Dpysl3	Acetyl [K367; K371; K503; K536]
Q9EQF6	Dihydropyrimidinase-related protein 5	Dpysl5	Acetyl [K13; K115; K504; K505; K546]
Q80Z24	Neuronal growth regulator 1	Negr1	Acetyl [K92; K209; K210]
E9PV14	Band 4.1-like protein 1	Epb41l1	Acetyl [K115; K259; K291; K498; K514]
P97300	Neuroplastin	Nptn	Acetyl [K226; K243; K259]
Q810U4	Neuronal cell adhesion molecule	Nrcam	Acetyl [K67; K529]
Q9CS84	Neurexin-1	Nrxn1	Acetyl [K451]
E9Q7×7	Neurexin-2	Nrxn2	Acetyl [K457]
Q6P9K9	Neurexin-3	Nrxn3	Acetyl [K411]
A0A0J9YUL3	Septin	Septin11	Acetyl [K170; K171; K184; K190; K195; K272; K315; K326; K336; K337; K397; K398; K418; K419]
D3Z5K8	SH3 and multiple ankyrin repeat domains protein 2	Shank2	Acetyl [K996]
P06837	Neuromodulin	Gap43	Acetyl [K30; K37; K80; K81; K199; K206; K208]
P03995	Glial fibrillary acidic protein	Gfap	Acetyl [K92; K276; K353; K402]
Q80YX1	Tenascin	Tnc	Acetyl [K2056]
P46660	Alpha-internexin	Ina	Acetyl [ 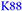 ; K111; 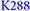 ; 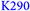 ; 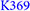 ; K447; K448]
E9Q0J5	Kinesin-like protein KIF21A	Kif21a	Acetyl [K671; K749]
P20917	Myelin-associated glycoprotein	Mag	Acetyl [ 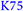 ; K575]
A0A668KLC6	Microtubule-associated protein	Map2	Acetyl [K615; K616; K621; K771; K838; K960; K1278; K1658; 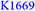 ; K1687; K1795; K1804; K1805; K1833; K1859; K1860; K1869; K1874]
Q3UH19	Microtubule-associated protein	Mapt	Acetyl [K243; K256; K269; K270; K287; K300; K306; K358; K359; K374]
Q542T4	Myelin basic protein	Mbp	Acetyl [K52; K57; K62; K83; K98; K115; K129]
Q9D2P8	Myelin-associated oligodendrocyte basic protein	Mobp	Acetyl [K36; K48; K55; K56; K67; K79; K95; K97; 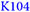 ; K113]
Q9WV34	MAGUK p55 subfamily member 2	Mpp2	Acetyl [K316; K317]
A0A0A6YY91	Neural cell adhesion molecule 1 (Fragment)	Ncam1	Acetyl [K75; K79; K251; K262; K268; K320; K501; K517; K612; K636; K658]
P19246	Neurofilament heavy polypeptide	Nefh	Acetyl [K435; K943]
P08551	Neurofilament light polypeptide	Nefl	Acetyl [ 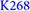 ; K272; 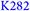 ; 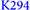 ]
P08553	Neurofilament medium polypeptide	Nefm	Acetyl [K53; K166; K261; K291; K296; K599; K602; K622; K704; K751; K788; K789; K807]
A0A087WPX3	Neurofascin	Nfasc	Acetyl [K123; K299; K534]
G3XA53	Oligodendrocyte-myelin glycoprotein	Omg	Acetyl [K145]
A0A0G2JFT8	Protein RUFY3	Rufy3	Acetyl [ 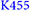 ; K470; K472]
O55042	Alpha-synuclein	Snca	Acetyl [K21; K43; K58; 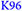 ; K97]
Q91ZZ3	Beta-synuclein	Sncb	Acetyl [K21; K43; K57; K84; K85]
P54227	Stathmin	Stmn1	Acetyl [K52; K53; 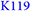 ; K128]
Q8BYI9	Tenascin-R	Tnr	Acetyl [K424; K643; K1301; K1327]
Q9CWF2	Tubulin beta-2B chain	Tubb2b	Acetyl [K19; 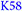 ; K103; K122; K174; K297; K336; K350; K379]
Q9ERD7	Tubulin beta-3 chain	Tubb3	Acetyl [K19; 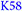 ; K122; K154; K174; K297; K336; K379]
**Synapse and vesicle transport**			
P17426	AP-2 complex subunit alpha-1	Ap2a1	Acetyl [K31; K35; K117; 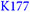 ; K378; K498; K905; K907]
Q5SWR1	AP complex subunit beta	Ap2b1	Acetyl [K26; 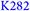 ; K283; 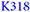 ; K322; K733; K735; K892; K931]
Q3TH69	AP-2 complex subunit mu	Ap2m1	Acetyl [ 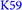 ; K139; 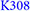 ; 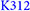 ; 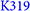 ; K339; K378; K379; 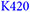 ]
Q3U8S0	Adaptor-related protein complex 3, sigma 1 subunit	Ap3s1	Acetyl [K41]
O88737	Protein bassoon	Bsn	Acetyl [K517; K761; K3702]
P84086	Complexin-2	Cplx2	Acetyl [K32; K33; K98; K99; K133; K134]
Q80TZ3	Putative tyrosine-protein phosphatase auxilin	Dnajc6	Acetyl [K139]
Q9QYX7	Protein piccolo	Pclo	Acetyl [K919]
Q03517	Secretogranin-2	Scg2	Acetyl [K575]
A2ALV3	Endophilin-A1	Sh3gl2	Acetyl [K28; K149; K159; K171; K172]
Q3UYK6	Amino acid transporter	Slc1a2	Acetyl [ 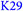 ; K157; K193; 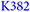 ; 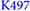 ; K525; K557; K569]
Q543U3	Amino acid transporter	Slc1a3	Acetyl [K191; K542]
Q69ZW4	MKIAA0899 protein (Fragment)	Ap2a2	Acetyl [K60; K64; K146; K406; K598; K660; K884]
E9QLK9	Clathrin coat assembly protein AP180	Snap91	Acetyl [K38; K39; 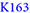 ; 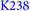 ; K785]
P63040	Complexin-1	Cplx1	Acetyl [K32; K133; K134]
Q8R1B5	Complexin-3	Cplx3	Acetyl [K84]
Q9QYS2	Metabotropic glutamate receptor 3	Grm3	Acetyl [K366]
G5E8D5	Metabotropic glutamate receptor 7	Grm7	Acetyl [K71]
Q497P1	Syntaxin 1A (Brain)	Stx1a	Acetyl [K55; K70; 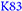 ; K84]
P61264	Syntaxin-1B	Stx1b	Acetyl [K45; 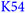 ; K55; K69; 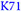 ; K82; K83; 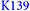 ]
O08599	Syntaxin-binding protein 1	Stxbp1	Acetyl [ 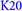 ; 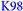 ; K120; K213; K225; 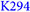 ; 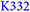 ; K356; K364; K493; 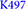 ; K524; 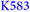 ; K584]
Q9JIS5	Synaptic vesicle glycoprotein 2A	Sv2a	Acetyl [K398; 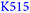 ]
Q8BG39	Synaptic vesicle glycoprotein 2B	Sv2b	Acetyl [K341; K424; K426; K465; K508]
B1AWV9	Anion exchange protein	Slc4a10	Acetyl [K286]
P60879	Synaptosomal-associated protein 25	Snap25	Acetyl [K69; K72; K94; K96]
Q69ZS6	Synaptic vesicle glycoprotein 2C	Sv2c	Acetyl [K506; K513]
O88935	Synapsin-1	Syn1	Acetyl [K128; 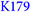 ; 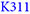 ; K324; K576]
Q64332	Synapsin-2	Syn2	Acetyl [ 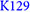 ; 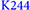 ; K442; K448]
Q80W45	Syntaxin-2	Stx2	Acetyl [K83; K84]
Q60770	Syntaxin-binding protein 3	Stxbp3	Acetyl [K357; K359]
Q62277	Synaptophysin	Syp	Acetyl [K89]
P46096	Synaptotagmin-1	Syt1	Acetyl [K189; K190; K191; K196; K222; K236; K272; K297; K321; K369; K420; K421]
A0A0R4J2C2	Synaptotagmin	Syt2	Acetyl [K190; K191; K192; K322]
E9Q3E2	Synaptopodin	Synpo	Acetyl [K586]
A0A498WGM0	Synaptotagmin-7	Syt7	Acetyl [K50]
B0QZN5	Synaptobrevin-2	Vamp2	Acetyl [K52; K83; K85]
Q80TB8	Synaptic vesicle membrane protein VAT-1 homolog-like	Vat1l	Acetyl [K238; K343; K344]
**Neurotransmitter transmission**			
P14231	Sodium/potassium-transporting ATPase subunit beta-2	Atp1b2	Acetyl [ 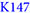 ; K276]
D5L240	Calcium-transporting ATPase	Atp2b2	Acetyl [K47; 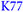 ; K363; K389; K500; K633; 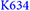 ; K784; K796; K829; K830; K944]
G3 × 9V4	Glutamate receptor	Grin2b	Acetyl [K221; K222]
P60521	Gamma-aminobutyric acid receptor-associated protein-like 2	Gabarapl2	Acetyl [K46]
Q80T41	Gamma-aminobutyric acid type B receptor subunit 2	Gabbr2	Acetyl [K854]
F6ZYH6	Gamma-aminobutyric acid receptor subunit beta-3	Gabrb3	Acetyl [K159]
C9K0Z0	Glutamate receptor	Gria2	Acetyl [K782; K850]
B0QZW1	Glutamate receptor	Gria3	Acetyl [K787; K861]
C9K0Y7	Glutamate receptor	Gria4	Acetyl [K783]
A2AI21	Glutamate receptor	Grin1	Acetyl [K207; K564; K565]
Q03717	Potassium voltage-gated channel subfamily B member 1	Kcnb1	Acetyl [K822]
Q91V14	Solute carrier family 12 member 5	Slc12a5	Acetyl [K691; K1085]
P31650	Sodium- and chloride-dependent GABA transporter 3	Slc6a11	Acetyl [K570; K590; K610; K612]
Q8K596	Sodium/calcium exchanger 2	Slc8a2	Acetyl [K348; K618]
P31648	Sodium- and chloride-dependent GABA transporter 1	Slc6a1	Acetyl [ 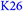 ; K28]

The blue represents the acetylated proteins and sites that have been reported in the rat brain acetylome (Lundby et al., 2012).

To validate the acetylation modification identified by MS analysis, we performed immunoprecipitation (IP) with anti-acetyllysine antibody conjugated beads, followed by immunoblot using commercially available antibodies against candidate proteins. The acetylated modification of three newly discovered acetylated brain-specific proteins was verified, including neurofilament light polypeptide (NEFL), 2’,3’-cyclic-nucleotide 3’-phosphodiesterase (CNP), and neuromodulin (GAP43) (Figure 7B).

## The cellular and subcellular specific distribution of acetylated proteins in the mouse brain

**FIGURE 8:**
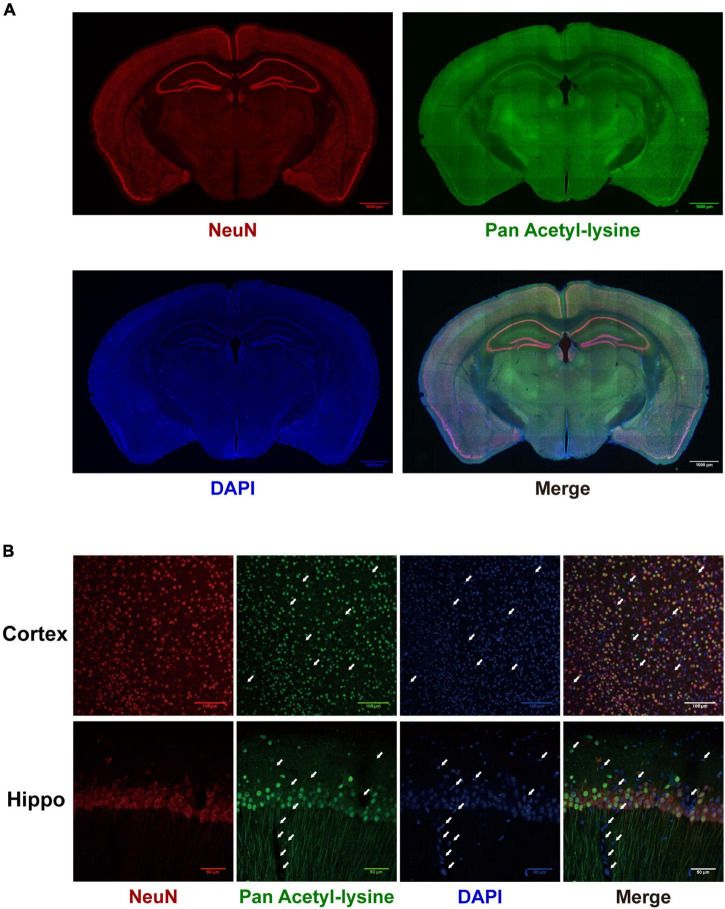
The distribution of acetylated proteins in the mouse brain. **(A)** The co-localization of acetylated proteins (Green) with the neuron (Red) was determined by immunofluorescence in coronary sections of the mouse brain. Scale bars = 1,000 μm. **(B)** Local magnification of cortex and hippocampus (*n* = 3). White arrows indicated the negatively stained nuclei. Scale bar = 50 μm.

**FIGURE 9:**
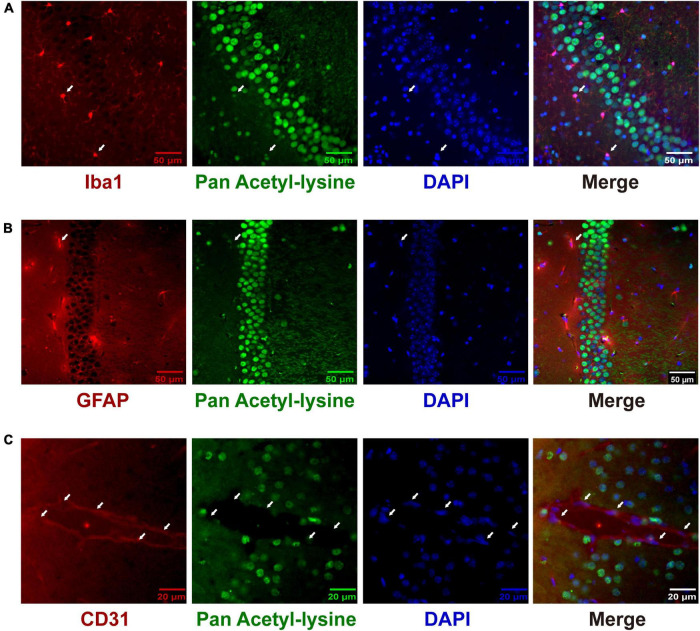
The distribution of acetylated proteins in microglia **(A)**, astrocytes **(B)**, and cerebrovascular endothelial cells **(C)**. The co-localization of acetylated proteins (Green) with microglia, astrocytes, and cerebrovascular endothelial cells (Red) was analyzed by immunofluorescence in coronary sections of the mouse brain. Representative hippocampus images of Iba1 **(A)** and GFAP **(B)** staining (*n* = 3); scale bar = 50 μm. Representative cortex image of CD31 **(C)** staining (*n* = 3); scale bar = 20 μm. The white arrows indicated the positively stained nuclei of Iba1- and GFAP-positive cells and the negatively stained nuclei of CD 31-positive cells. Scale bar = 50 or 20 μm.

Although many studies have investigated the roles and mechanisms of protein acetylation in brain development and neurological disorders, the cellular distribution of the acetylated proteins in the brain is still unclear. A panoramic view of the immunofluorescence-stained coronal section illustrated the broad and specific distribution of acetylated proteins in the mouse brain (Figure 8A). NeuN is a marker for neurons. The positive staining of acetylated proteins was mainly colocalized with NeuN staining in the cortex and hippocampus (Figure 8A, the full-size images were provided in Supplementary Figures 1, 2). Local magnification indicated that the positive staining for acetylated proteins was mainly colocalized with nuclei (DAPI) of the NeuN positive cells in the cortex (Figure 8B) and positive fibrous staining in the axons of hippocampal neurons (Figure 8B). IBA1 and GFAP are markers of microglia and astrocytes, respectively. Compared with the neurons, only a tiny portion of microglia and astrocyte nuclei (Figures 9A,B) were positively stained. Notably, some nuclei in the brain were negatively stained for acetylated proteins, 7.32 ± 1.36% and 10.01 ± 1.82% in the cortex and hippocampus, respectively (Figure 8B). The further experiment demonstrated that these negatively stained nuclei and cytoplasm were colocalized with CD31 positive stained endothelial cells (Figure 9C). In short, these results illustrated the cellular and subcellular specific distribution of acetylated proteins in the mouse brain.

## Discussion

As an essential step toward understanding the mechanism underlying the multiple roles of protein acetylation in brain development and various neurological disorders, we profiled and characterized the mouse brain acetylome. Using a simplified acetylome profiling strategy, we identified 1,818 acetylated proteins containing 5,196 acetylation modification sites in C57BL/6 mice brains. To our knowledge, this is the most comprehensive mouse brain acetylome. Bioinformatics analysis highlighted the tissue specificity of the brain acetylome. Manual annotation revealed that a set of brain-specific proteins were acetylation modified. Further immunofluorescence staining illustrated the cellular-specific distribution of brain acetylated proteins. Notably, an unexpected finding was the significant differences between the present mouse brain acetylome and the previously reported rat brain acetylome (Lundby et al., 2012).

### Profiling a comprehensive mouse brain acetylome using a simplified workflow

Usually, the sample processing steps for acetylome profiling include proteolysis, acetylation peptide enrichment, and peptide fraction by SCX/high pH HPLC before or after enrichment (Svinkina et al., 2015; Diallo et al., 2019). Acetylation is a widespread posttranslational modification but has a low probability of occurrence. Stoichiometric analysis of acetylated proteins in Hela cells revealed that most acetylation occurs at very low stoichiometry (median 0.02%) (Hansen et al., 2019). Thus, most acetylated peptides are below the detection threshold without antibody enrichment. As antibody affinity chromatography can dramatically reduce the sample complexity, in this study, we tried a simplified workflow for analyzing acetylome, comprising FASP, acetylated peptides enrichment, and mass spectrometry analysis without pre- or post-fraction. In this case, we identified 1,818 acetylated proteins containing 5,196 acetylation modification sites in C57BL/6 mice brains. The coverage of this mouse acetylome was even higher than that of the rat brain acetylome using a post SCX fraction (3 fractions, 1,653 acetylated proteins with 4,782 modification sites) (Lundby et al., 2012), and the mouse brain acetylome using a pre SCX fraction (6 fractions, 523 acetylated proteins with 1,247 modification sites) (Dittenhafer-Reed et al., 2015). This simplified workflow dramatically reduces the labor and time for sample preparation, MS analysis, and subsequent data processing without sacrificing the coverage of acetylated sites, peptides, and proteins. Combined with the label or label-free quantitative proteomic strategies, this simplified workflow will be more suitable for identifying the pathophysiological relevant acetylated proteins in brain development and neurological disorders.

### The tissue specificity of the mouse brain acetylome and the acetylated brain-specific proteins

Corroborated the notion of organ specificity of lysine acetylation (Lundby et al., 2012), this study manifested the tissue specificity of the mouse brain acetylome. Firstly, WB results directly illustrated the distinct expression pattern of acetylated proteins between the brain and other tissues. Secondly, GO and KEGG analyses on the mouse brain and liver acetylome showed significant differences in their enrichment in the cellular compartment and biological pathways. Thirdly, manual annotation further revealed that many brain-specific or highly expressed proteins were acetylated, including cytoskeletal, synaptic, and ion channel proteins. In addition to the tissue specificity of protein expression, the tissue specificity of acetylome could be related to differences in the regulation of protein acetylation in different tissues, such as the difference in the expression and distribution of HATs and HDACs in different tissues (Supplementary Figure 3A). This issue is worthy of further exploration.

So far, most studies on the roles of protein acetylation in brain development have focused on the epigenetic mechanisms mediated by histone acetylation (D’mello, 2019). Recent studies have started to explore the biological functions of these acetylated non-histone proteins in CNS development and disorders, such as the acetylation of alpha-tubulin (Cho and Cavalli, 2012; Dan et al., 2018), AMPA Receptor (Wang et al., 2017), and Tau (Shin et al., 2021). Analyzing the dynamics of these non-histone proteins’ acetylations under different physiopathological conditions and investigating the biological consequences of the acetylation of these non-histone proteins will extend our understanding of the roles and mechanisms of protein acetylation in brain development and neurological disorders.

### The cellular-specific distribution of acetylated proteins in the mouse brain

An interesting finding of this study is the cellular and subcellular specific distribution of acetylated proteins in the mouse brain. Like the distribution of HDACs in the brain (Broide et al., 2007), acetylated brain proteins are mainly distributed in neurons. Besides the nuclei, the acetylated proteins were predominantly located in the axons of hippocampal neurons. Corroborated this subcellular-specific distribution, our manual annotation revealed that many neuron-specific cytoskeleton proteins are modified by acetylation, such as the multiple isoforms of Neurofilament (Table 2).

Histones are the main protein components of the nucleus. Histone acetylation is amarker ofactive gene transcription (Jenuwein and Allis, 2001). Interestingly, we found that both the nuclei and cytoplasm of cerebrovascular endothelial cells lacked the distribution of acetylated proteins. It has been reported that in adulthood and under quiescent conditions ECs are quiescent with a long turnover time, and the net turnover rate of BMECs is expected to approach zero (Destefano et al., 2018). Thus, the hypoacetylation of the nucleus of cerebrovascular endothelial cells might reflect the low proliferation and metabolism of cerebrovascular endothelial cells.

Given the nature of the cell-specific distribution of acetylated brain proteins (Supplementary Figure 3B), the cellular context of the protein acetylation’s biological roles should be considered when studying the roles and mechanisms underlying the effects of HAT and HDACs mutation or HDACis on brain development, function, and neurological disorders.

### The significant differences between the acetylomes of mouse and rat brain

An unexpected finding of this study is the significant differences observed between the present mouse brain acetylome and the previous rat brain acetylome (Lundby et al., 2012). Firstly, although the number of acetylation proteins and sites is similar between these two brain acetylomes, the overlapped proteins and sites are very small. Secondly, the subcellular distribution of brain acetylome is different. The present data showed that acetylated proteins of the mouse brain were mainly distributed in the cytoskeleton, while those of the rat brain were mainly distributed in the plasma membrane. What accounts for this discrepancy?

The results of the Motif analysis provided us with some clues to understanding this discrepancy. Motif analysis indicated that the sequence specificity of acetylated motifs in the rat brain was more similar to that of the mouse liver rather than the mouse brain. This discrepancy most likely reflects the preference of sequences recognized by antibodies for acetylated peptide enrichment (Supplementary Figure 4). Immunechem antibody (ICP0388) was used to profile the acetylome of rat brain and mouse liver (Lundby et al., 2012; Weinert et al., 2013), while PTM antibody (PTM104) was used in the present study. Besides, the results of acetylome profiling could also be affected by the different protein extraction and enzymatic digestion methods. In this study, we replaced frequently used in-solution digestion with FASP. Compared with in-solution digestion, the peptides resulting from FASP have a more uniform length (Wisniewski et al., 2009), which could help improve the efficiency and coverage of acetylated peptide identification. (Supplementary Table 7

Acetylome analysis is a powerful tool for understanding and exploring the role and mechanism of protein acetylation in brain development, function, and nervous system diseases. However, research in this area is still in its infancy, and many problems remain to be solved. As the significant differences observed between mouse and rat brain acetylomes, a standardized workflow for profiling acetylomes is needed. Thus data from different acetylome studies from different groups would be comparable.

## Conclusion

Together, this study provided and characterized a comprehensive mouse brain acetylome and demonstrated the cellular-specific distribution of brain acetylated proteins. Both the dataset and the simplified workflow will contribute to further exploring the molecular and cellular mechanisms of protein acetylation in brain development, functions, and various neurological disorders.

## Data availability statement

The data supporting this study’s findings are available in ProteomeXchange Consortium at http://www.proteomexchange.org, reference number PXD034594.

## Ethics statement

The animal study was reviewed and approved by the Administration Committee of Experimental Animals of Jinan University.

## Author contributions

YJ, JJ, and QJ designed experiments and drafted the manuscript. ZXC, XL, ZQC, and YY performed proteomic experiments and bioinformatics analyses. SL, QS, and DW performed immunostaining experiments. All authors contributed to the article and approved the submitted version.

## Supplementary material

Supplementary Figure 1 The full-size immunostaining images of the distribution of acetylated proteins in the mouse brain.

Supplementary Figure 2 The negative controls of the immunostaining.

Supplementary Figure 3 The expression of HATs and HDACs in different tissues and brain cells.

Supplementary Figure 4 The comparison of the antibodies against acetylated lysine provided by different vendors using immunostaining.

Supplementary Table 1 Peptides identified from enriched samples.

Supplementary Table 2 Peptides and proteins identified from purified samples.

Supplementary Table 3 Identified acetylated sites and proteins.

Supplementary Table 4 Mapping the identified acetylated sites and proteins to known databases.

Supplementary Table 5 Rank of 1170 overlapped proteins and acetylated proteins.

Supplementary Table 6 Manual annotation of mouse brain acetylome.

Supplementary Table 7 Comparison of the workflows in the current and a previous study.
