# Supplementary figures and images for: A comprehensive mouse brain acetylome-the cellular-specific distribution of acetylated brain proteins
Source: Front Cell Neurosci. 2022 Aug 30;16:980815. doi: 10.3389/fncel.2022.980815 (PMC9468461; doi:10.3389/fncel.2022.980815)

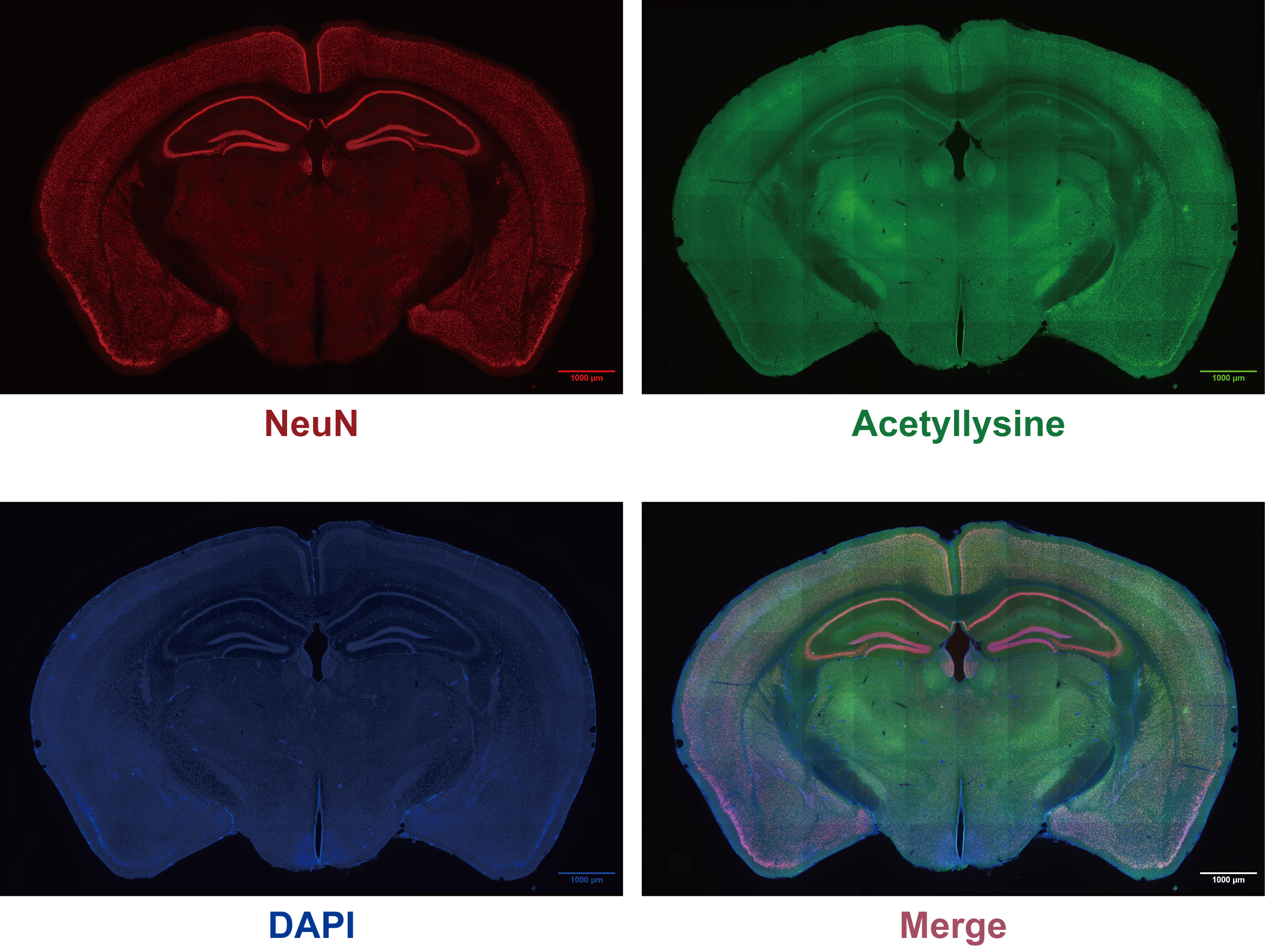

Supplement: Supplementary Figure 1 — The full-size immunostaining images of the distribution of acetylated proteins in the mouse brain. [file Image_1.tif]

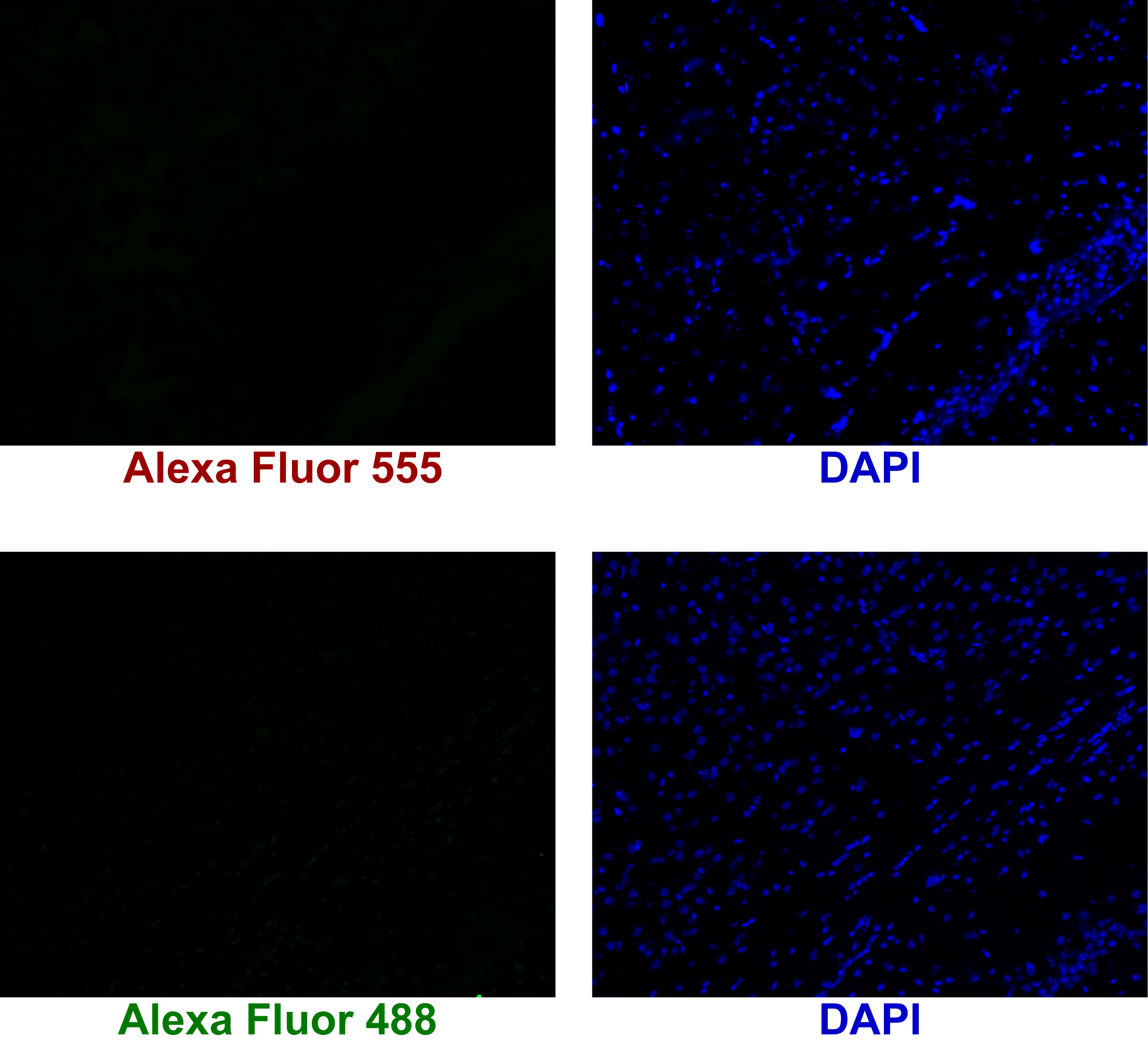

Supplement: Supplementary Figure 2 — The negative controls of the immunostaining. [file Image_2.tif]

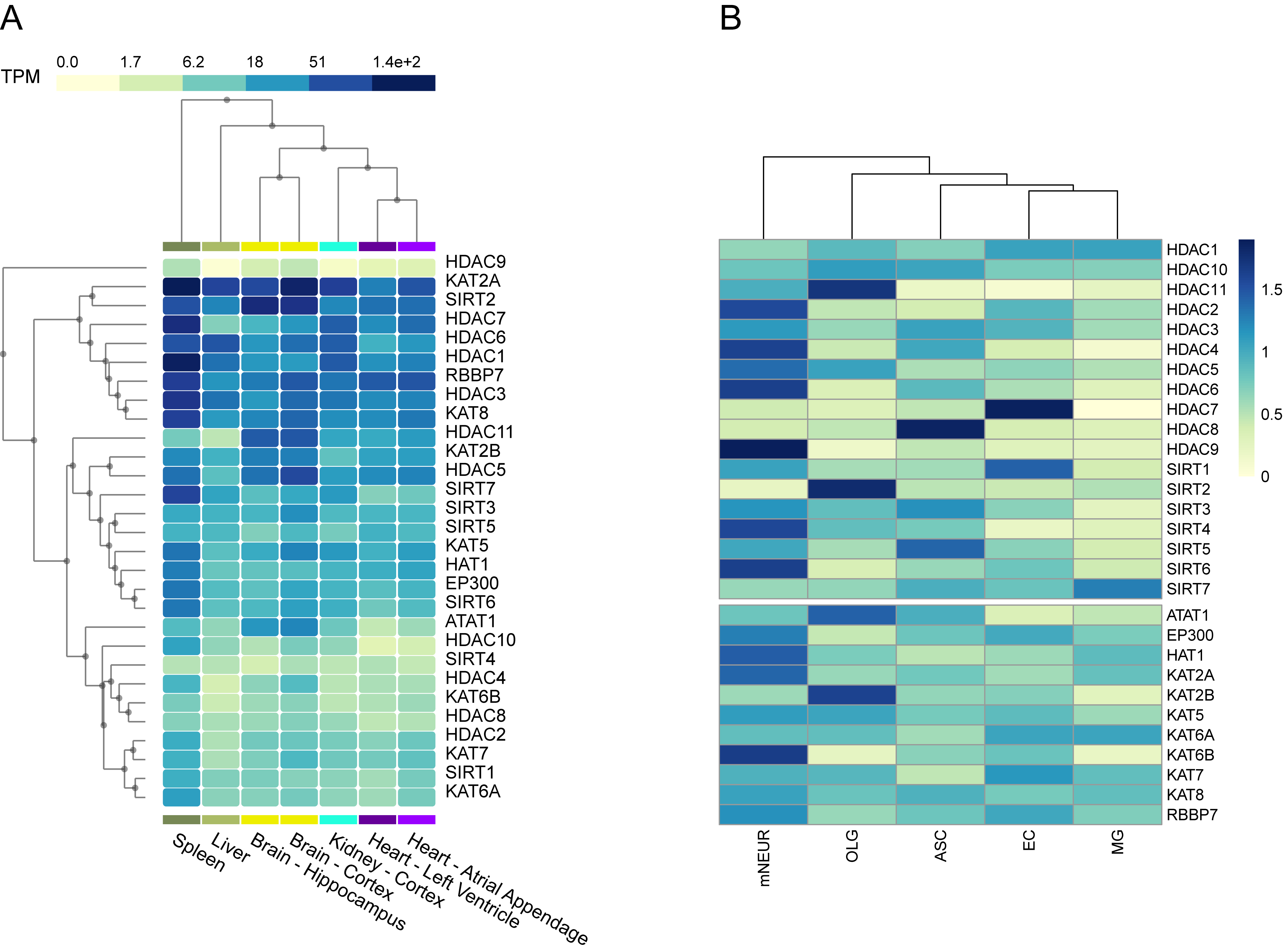

Supplement: Supplementary Figure 3 — The expression of HATs and HDACs in different tissues and brain cells. [file Image_3.tif]

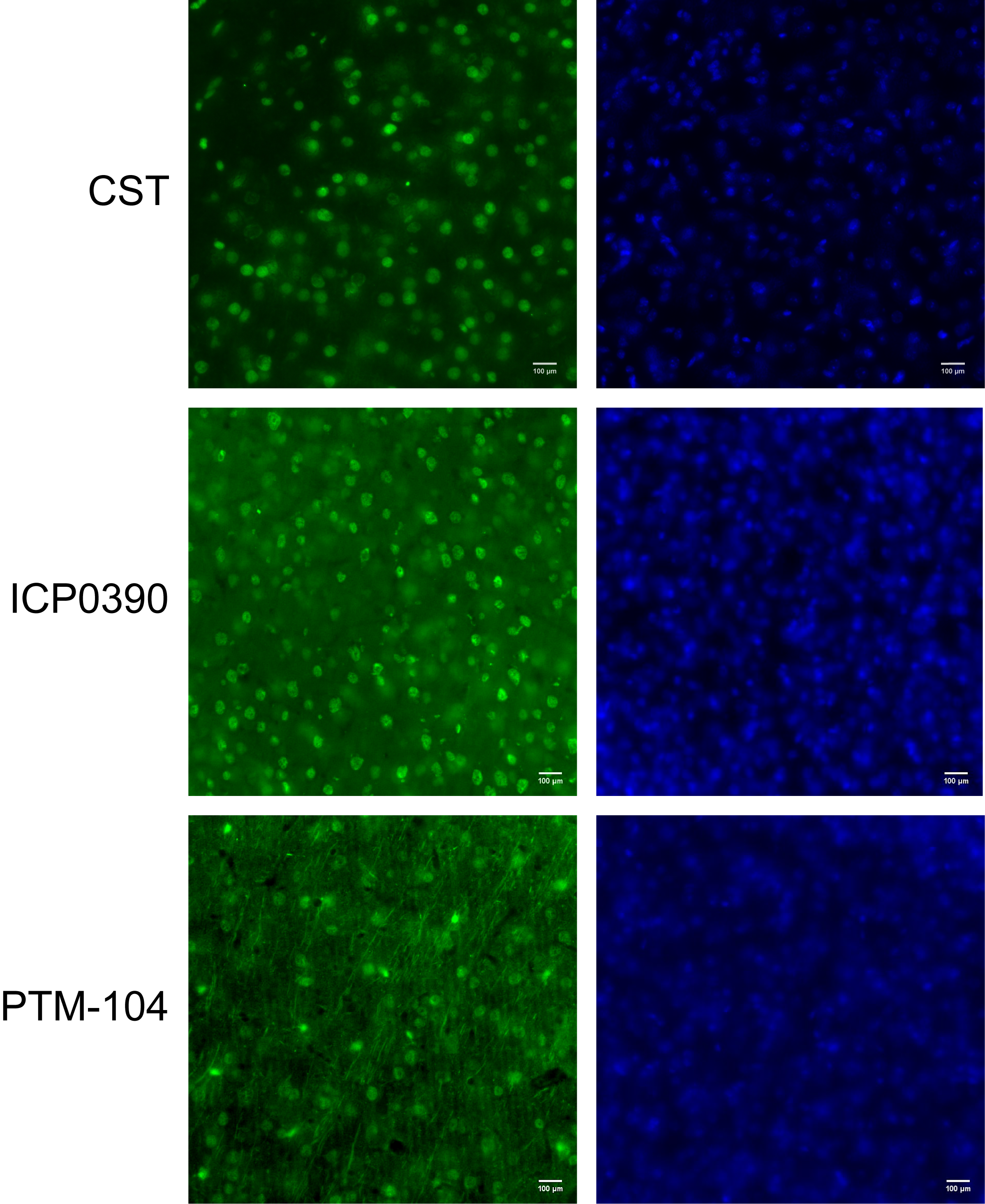

Supplement: Supplementary Figure 4 — The comparison of the antibodies against acetylated lysine provided by different vendors using immunostaining. [file Image_4.tif]
